# Bone scintigraphy elucidates different metabolic stages of melorheostosis

**Published:** 2012-02-13

**Authors:** Sina Izadyar, Ali Gholamrezanezhad

**Affiliations:** 1 Department of Nuclear Medicine. Imam Khomeini Complex Hospitals. Tehran University of Medical Sciences, Tehran, Iran

**Keywords:** Melorheostosis, 99mTechnetium-MDP, Bone Scintigraphy, Iran

## Abstract

Melorheostosis is a rare benign non-hereditary sclerosing dysplasia involving the bone, often in a sclerotomal distribution. we report the case of a 27 years old lady with painful swelling of the left hand and forearm lasting for almost 15 years. The patient experienced aggravation of symptoms and limitation of motion during the past two months. Radiographic assessment revealed hyperostosis involving the left 3^rd^ and 4^th^ metacarpal bones and corresponding digits as well as the left ulna and distal humerus, with no soft tissue ossification. Angiographic and blood pool images of bone scintigraphy showed increased activity of mid-metacarpal region, corresponding to the sclerotom C-8. Delayed static views showed increased radiotracer uptake of the left 4^th^ metacarpal bone and the corresponding digit as well as the left ulna and humerus, but no abnormal osteoblastic activity of the 3^rd^ left metacarpal and digit. Histopathologic assessment confirmed the diagnosis of Melorheostosis. The case confirms that even in the same sclerotomal distribution, the multiple foci of involvement can present in different metabolic stages. In fact, the disease does not progress uniformly and different lesions can be seen in dissimilar stages of activity. Hence, metabolic imaging can be important to unmask which of the radiographically detected bony lesions are metabolically active and have the potential to be the source of current patient's symptoms and which of them are old, metabolically inactive and silent lesions, which are not clinically relevant to the patient's complaints.

## Introduction

Melorheostosis is a rare benign non-hereditary sclerosing dysplasia involving the bone, often in a sclerotomal distribution [1]. The disease is evident by age of 20 in almost half of the cases and affects both sexes equally [2]. Although genetic predisposition [2], anomalies of blood vessels or lymph vessels [3], and metabolic abnormalities [4] have been proposed as possible underlying pathophysiologic causes, the exact etiology of Melorheostosis is yet to be determined.

The role of clinical imaging modalities, including magnetic resonance imaging and computed tomography in revealing the spectrum of the disease manifestation and differentiation from other possible diagnoses and excluding malignancy has been emphasized [2]. Bone scintigraphy is one of the most useful imaging modalities to assess the metabolic activity of the skeletal lesions [5]. In this regard, the application of bone scintigraphy in patients suspicious for Melorheostosis has been reported [1–2].

## Case Report

**Figure 1:**
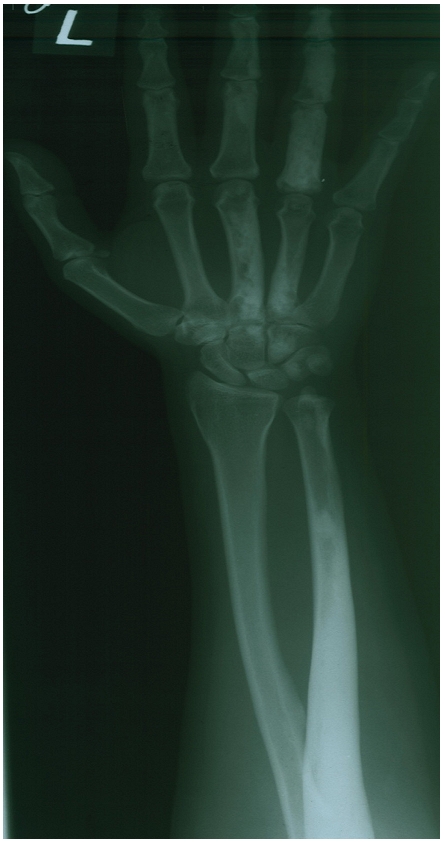
Plane radiographic images showing hyperostosis of the 3rd and 4th metacarpal and digits as well as ulna and distal humerus

**Figure 2:**
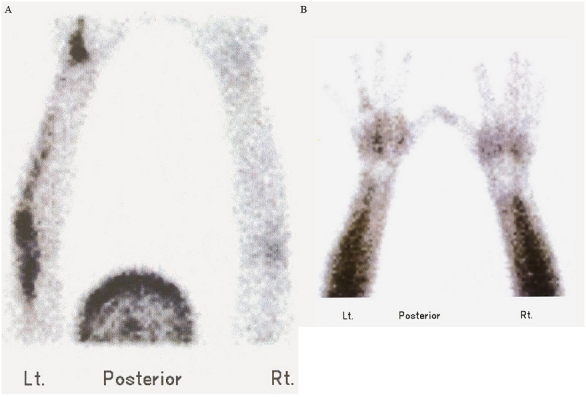
Angiographic (A) and blood pool (B) images using 99mTc-MDP, showed hyperemia involving mid-metacarpal region

**Figure 3:**
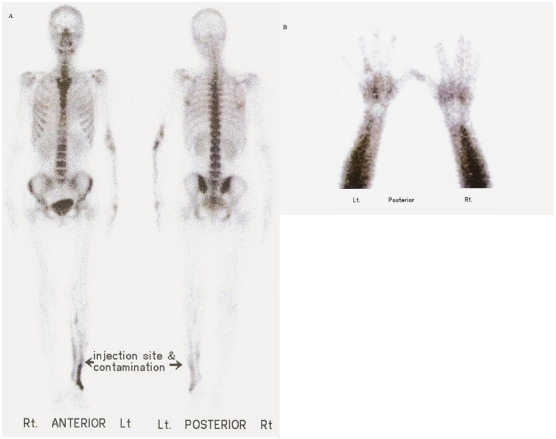
Static delayed views of 99mTc-MDP bone scintigraphy [whole body (A), and hand posterior spot view (B)], showing bone involvement in different stages of metabolic activity

Our case was a 27 year old lady with painful swelling of the left hand and forearm lasting for almost 15 years. The patient experienced aggravation of symptoms and limitation of motion during the past two months. Radiographic assessment revealed hyperostosis involving the left 3^rd^ and 4^th^ metacarpal bones and corresponding digits as well as the left ulna and distal humerus, with no soft tissue ossification (Figure 1). Early angiographic and blood pool images of bone scintigraphy with 20mCi (740MBq) ^99m^Tc-MDP showed increased activity of mid-metacarpal region, corresponding to the sclerotom C-8 (Figure 2). Delayed static views showed increased radiotracer uptake of the left 4^th^ metacarpal bone and the corresponding digit as well as the left ulna and humerus, but no abnormal osteoblastic activity of the 3^rd^ left metacarpal and digit (Figure 3). Subsequently, histopathologic assessment confirmed the diagnosis of Melorheostosis.

Two main features can be found with our case. The first one is the involvement of multiple bony structures in the upper extremity, which is not a common feature of the disease. The primary sites of involvement are long bones of the lower limbs and just few cases of the disease affecting the upper extremities have been reported [1]. More importantly, the case confirms that even in the same sclerotomal distribution, the multiple foci of involvement can present in different metabolic stages. In fact, the disease does not progress uniformly and different lesions can be seen in dissimilar stages of activity. Hence, metabolic imaging can be important to unmask which of the radiographically detected bony lesions are metabolically active and have the potential to be the source of current patient's symptoms and which of them are old, metabolically inactive and silent lesions, which are not clinically relevant to the patient's complaints.

## Conclusion

Whole body scan using bone seeking radiopharmaceuticals is a valuable tool for the assessment of patients with developmental and metabolic bone disorders.
